# International Health Regulations core capacities monitoring in 2020-2021 at Italian Points of Entry

**DOI:** 10.1093/eurpub/ckac131.409

**Published:** 2022-10-25

**Authors:** E Frisicale, S Zichichi, A Di Virginio, R Falvo, U Angeloni, G Rezza

**Affiliations:** 1 Directorate General of Health Prevention, Ministry of Health, Rome, Italy; 2 Cross Border Health Authorities, Ministry of Health, Palermo, Italy; 3 School of Hygiene and Preventive Medicine, University of Perugia, Perugia, Italy; 4 Committee of Palermo, Italian Red Cross, Palermo, Italy

## Abstract

Under the International Health Regulations (IHR) 2005 States Parties (SP) are required to develop and maintain core public health capacities and report related progress to World Health Organization (WHO) annually,including those related to Points of Entry (PoEs).Using an appropriate tool,provided by WHO, Italian PoEs assessments in 2020 and 2021 were conducted to have the status quo,describe any differences among years and promote corrective actions if required. The tool was designed in 2009 to support SP in ascertaining prior capacities in PoEs and monitoring their progress in order to designate PoEs under IHR.The tool was translated in Italian.Coordination and communication among authorities involved in each PoE (C1),core capacity at all times (C2) and at PHEIC (Public Health Emergency of International Concern) (C3) were assessed by cross border health Authorities for 2020 and 2021.For each of these sections the maximum score is 100%. For each year,a descriptive analysis was conducted and any differences in scores between the two years were noted.The presence of a contingency and vectors’ control plans was also investigated.The assessment was completed respectively for 2020 and 2021 by 24 and 25 airports, 53 and 52 ports. C1 in 2020 resulted on average 89% for airports and 95% for ports, in 2021 respectively 90% and 95%; C2 in 2020 resulted on average 64% for airports and 63% for ports, in 2021 respectively 63% and 64%; C3 in 2020 resulted on average 68% for airports and 61% for ports,in 2021 respectively 63% and 58%.Few differences in scores between 2020 and 2021 were noted in some PoEs. 4 PoEs declared the presence of a contingency plan only in 2021.Few PoEs stated to not have a vectors’ control plan in both years.The assessment pointed out a good level of coordination and communication among all the stakeholders involved, including the Italian Red Cross,but it revealed lower scores for the other IHR core capacities. Improvement actions have to be pursued surely.

**Key messages:**

• The specified tool helps cross border and national Authorities to understand strengths and weaknesses in each PoE and leads to develop a specific plan of action to overcome potential gaps.

• Maintaining a good level of core capacities at points of entry is fundamental to protect national public health.

